# Emerging Anti-Diabetic Drugs for Beta-Cell Protection in Type 1 Diabetes

**DOI:** 10.3390/cells12111472

**Published:** 2023-05-25

**Authors:** Nida Ajmal, Maislin C. Bogart, Palwasha Khan, Ibiagbani M. Max-Harry, Craig S. Nunemaker

**Affiliations:** 1 Department of Biomedical Sciences, Heritage College of Osteopathic Medicine, Ohio University, Athens, OH 45701, USA; na816922@ohio.edu (N.A.); ps831721@ohio.edu (P.K.); im830219@ohio.edu (I.M.M.-H.); 2 Translational Biomedical Sciences Graduate Program, Ohio University, Athens, OH 45701, USA; 3 Honors Tutorial College, Ohio University, Athens, OH 45701, USA; mb758718@ohio.edu; 4 Molecular and Cellular Biology Graduate Program, Ohio University, Athens, OH 45701, USA

**Keywords:** type 1 diabetes, beta cells, hyperglycemia, investigational new drugs

## Abstract

Type 1 diabetes (T1D) is a chronic autoimmune disorder that damages beta cells in the pancreatic islets of Langerhans and results in hyperglycemia due to the loss of insulin. Exogenous insulin therapy can save lives but does not halt disease progression. Thus, an effective therapy may require beta-cell restoration and suppression of the autoimmune response. However, currently, there are no treatment options available that can halt T1D. Within the National Clinical Trial (NCT) database, a vast majority of over 3000 trials to treat T1D are devoted to insulin therapy. This review focuses on non-insulin pharmacological therapies. Many investigational new drugs fall under the category of immunomodulators, such as the recently FDA-approved CD-3 monoclonal antibody teplizumab. Four intriguing candidate drugs fall outside the category of immunomodulators, which are the focus of this review. Specifically, we discuss several non-immunomodulators that may have more direct action on beta cells, such as verapamil (a voltage-dependent calcium channel blocker), gamma aminobutyric acid (GABA, a major neurotransmitter with effects on beta cells), tauroursodeoxycholic acid (TUDCA, an endoplasmic reticulum chaperone), and volagidemab (a glucagon receptor antagonist). These emerging anti-diabetic drugs are expected to provide promising results in both beta-cell restoration and in suppressing cytokine-derived inflammation.

## 1. Introduction

Type 1 diabetes (T1D) is a chronic autoimmune disorder that occurs due to the destruction of the beta cells present in the islets of Langerhans of the pancreas. Autoreactive T lymphocytes are responsible for the destruction of insulin-producing beta cells, which results in the severe impairment of blood glucose regulation [1]. In addition to hyperglycemia complications, there is a likelihood of other serious complications that are associated with T1D, such as diabetic hyperosmolar syndrome or diabetic ketoacidosis (DKA) [1]. Moreover, individuals with type 1 diabetes are also susceptible to other conditions, such as celiac disease, vitiligo, Addison’s disease, and thyroid autoimmunity [2].

There is no cure available that prevents the destruction of beta cells. Presently, T1D is managed by the administration of exogenous insulin, either through subcutaneous injections or by an insulin pump that also includes continuous glucose monitoring (4.0–7.8 mmol/L) [3]. Lack of insulin, being a hallmark of T1D, is a lifelong struggle. This way of managing T1D is sometimes difficult and costly. If the glucose level is not controlled via insulin administration, it will lead to life-threatening conditions, such as diabetic ketoacidosis. Hence, there is a dire need for optimal therapy that restores and retains beta-cell function and insulin production to regulate blood glucose levels.

T1D therapy should be designed to reduce the risk of other metabolic conditions associated with it, such as hypoglycemia, hyperglycemia, and ketoacidosis [4]. If poorly managed, T1D can lead to complications including heart disease, kidney disorders, nerve damage, and other problems with feet, oral health, vision, hearing, and mental health. Recent advances in T1D therapeutics have focused on drugs that could protect beta cells and stimulate insulin secretion. For this reason, several existing medications to treat type 2 diabetes, such as dipeptidyl peptidase-4 inhibitors, glucagon-like peptide-1 receptor agonists, and sodium-glucose cotransporter-2 inhibitors, are being tested in subjects with T1D. We note that there is therapeutic potential in these drugs to protect beta cells and enhance their function, however, we considered these well-studied T2D medications to be outside the scope of this review. This review focuses on novel investigational new drugs (INDs) that aim, at least in part, to directly protect beta cells.

## 2. Methods: A Description of Search Parameters, Results, and Choices Made for Exclusion or Inclusion

ClinicalTrials.gov was searched for clinical trials of drugs associated with the treatment of T1D using the single search term “type 1 diabetes” as a condition on 8 March 2023. There were 3250 hits, the vast majority of which were related to insulin therapy or repurposed type 2 diabetes medications that were excluded as outside the scope of this review. Similarly, we identified 40 unique immunomodulatory drugs with 212 associated clinical trials that were also excluded. Among the remaining trials, we identified four emerging INDs that appear to have direct effects on the beta cells. These four INDs are the focus of this review.

## 3. Investigational New Drugs with Effects on Beta Cells

Although immunomodulators can aid in protecting beta cells and reversing the immune response, overall, this approach has not been successful in reversing T1D complications [5]. The infiltration of pancreatic islets by T lymphocytes, macrophages, and other immune cells causes damage to the beta cells [6]. These immunological abnormalities also include autoantibodies such as the 65KD isoform of glutamic acid decarboxylase (GAD65), tyrosine phosphatase-related islet antigen 2 (IA2 or ICA512), zinc transporter (ZnT8), and regulatory T cell (Treg) alterations, that suppress the action of effector T cells [7]. Many ongoing studies are focused on suppressing the autoimmune response, but this can cause adverse effects and has been found to be effective for a limited duration in delaying the onset of T1D [7]. An alternative approach that is gaining support is to target the beta cell itself [8,9].

In searching the National Clinical Trials database, we identified four different investigational drugs that putatively target the pancreatic beta cells for at least some aspects of their therapeutic effects: verapamil (a voltage-dependent calcium channel blocker), gamma aminobutyric acid (GABA, a major neurotransmitter with effects on beta cells), tauroursodeoxycholic acid (TUDCA, an endoplasmic reticulum chaperone), and volagidemab (a glucagon receptor antagonist). Table 1 describes the presumed target and key effects on the beta cells for each of these pipeline therapeutics. Table 1 also includes a list of clinical trials to treat T1D for each drug.

### 3.1. Verapamil

Verapamil was the first non-dihydropyridine calcium channel blocker (CCB) approved by the FDA in 1981 for clinical use in the prevention of clinical implications such as cardiac arrhythmias and hypertension conditions [10]. By reducing calcium influx primarily through L-type voltage-gated calcium channels in the myocardium and vascular smooth muscle, verapamil promotes vasodilation to reduce hypertension.

**Figure 1:**
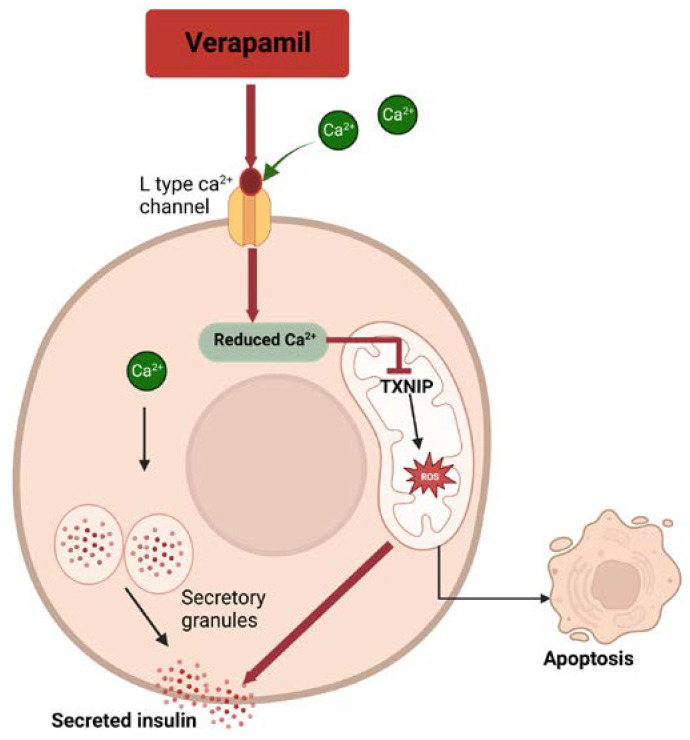
Verapamil, a calcium channel blocker, reduces intracellular calcium and inhibits mitochondrial TXNIP activity to prevent apoptosis of the beta cells.

L-type calcium channels also play a pivotal role in beta-cell function. As shown in Figure 1, calcium is a key element in the triggering pathway of insulin secretion. Following the entry of glucose through facilitated transport and glucose metabolism, the subsequent increase in ATP/ADP causes closure of ATP-gated potassium (KATP) channels and a depolarization in beta cells that triggers calcium influx through voltage-gated calcium channels [11]. This calcium influx directly triggers the release of insulin from granules through calcium-dependent exocytosis. Verapamil and other CCBs reduce glucose-stimulated insulin release by reducing calcium influx at the level of pancreatic beta cells [11].

Verapamil appears to have more complex effects in vivo. One of the first studies of glucose regulation following its FDA approval reported that verapamil reduced blood glucose in healthy fasted subjects [12]. Since participants were fasted, insulin secretion would be minimal with or without verapamil present. The authors speculated that reduced cytosolic calcium in hepatocytes could cause a decrease in gluconeogenesis, resulting in reduced blood glucose [12]. Subsequent studies in rats showed that verapamil could correct metabolic dysfunction in pancreatic islets by reducing intracellular calcium to improve mitochondrial activity and ATP production [13]. Our own work similarly showed that reducing glycolytic activity decreased intracellular calcium in islets from diabetic mice while paradoxically increasing ATP content and insulin secretion [14].

Another important feature of verapamil is that it is effective in reducing the expression of thioredoxin-interacting protein (TXNIP) in beta cells, which helps in the survival of beta cells during T1D. TXNIP is a cellular redox regulator that was identified as the top-most glucose-induced gene reported in a microarray study of glucotoxicity in human islets [15]. During T1D, TXNIP is upregulated in the beta cells, and this facilitates the mitochondrial death pathway [16]. TXNIP expression, when reduced, promotes beta-cell mass and survival and protects against diabetes [17]. Beta-cell destruction occurs because of the cytokines IL-1β, TNF-α, and IFN-ϒ, and it has been hypothesized that these cytokines are involved in the mitochondrial death pathway that also increases the expression of TXNIP [18]. A study revealed that among these three cytokines, IFN-ϒ particularly increased the expression of mRNA for TXNIP, while the other two cytokines have different roles in TXNIP expression [19].

Together, these findings show that TXNIP is a key target to treat and potentially reduce the risk of T1D. Calcium channel blockers, particularly verapamil, are effective in reducing the expression of TXNIP [20] (see Figure 1). Of note, calcium plays a central role in cytokine action as well as in the mitochondrial death pathway related to the mitochondrial permeability transition pore [21,22], which may or may not be directly linked with the effects of verapamil on TXNIP. For additional reading, a review by Zimmerman et al. provides a detailed description of verapamil with regard to calcium, TXNIP action, and diabetes [23].

Regarding the potential to treat T1D, verapamil has shown some promising results. The efficacy of verapamil was tested along with insulin intake in a randomized, double-blind, placebo-controlled phase 2 clinical trial (NCT02372253) in subjects with T1D. It revealed improved meal-stimulated C-peptide area under the curve levels as a measure of remaining beta-cell function and improved glycemic control. Constipation was the only side effect observed in the verapamil-treated group with no hypotension. The study found that verapamil is a potentially beneficial drug for newly diagnosed T1D individuals and promotes beta-cell function, delays beta-cell loss, and helps reduce insulin requirements and hypoglycemic episodes [24]. A follow-up study revealed that these beneficial effects last for at least two years while patients continue to take verapamil and that verapamil also has immunomodulatory effects [25].

A phase 3 clinical trial (NCT04233034) that involved testing verapamil along with insulin intake to assess the beta-cell preservation among T1D-diagnosed individuals was recently published [26]. In this randomized, double-blind trial of 88 children (age 7–17) with newly diagnosed T1D, beta-cell function during a mixed-meal tolerance test was 30% higher with verapamil compared with a placebo, as measured by the C-peptide level at 52 weeks from diagnosis. Verapamil was also well-tolerated by participants. The results from this phase 3 clinical trial suggest that verapamil protects beta-cell function; however, further study is needed to determine whether verapamil can maintain the observed improvements in C-peptide levels in the long term.

Additional clinical trials are underway. A randomized, double-blind, placebo-controlled phase 2 clinical trial (NCT04545151), along with testing insulin intake, is also currently recruiting, with the aim to confirm the effect of 360 mg of oral administration of verapamil alone and in combination with other drugs among participants with newly diagnosed T1D. The study is expected to be completed in February 2024. Another arm of this phase 2 clinical trial (NCT04615910) will assess the effect of verapamil on beta-cell mass in T1D individuals using 68-GA-NODAGA exendin, which is a radiolabeled GLP-receptor agonist that should help identify beta cells in vivo by positron emission tomography (PET) [27]. This clinical trial recorded information about insulin intake, but there is not enough information about insulin measurement as the clinical trial is still in the recruiting stage. The increase in the beta-cell mass causes the uptake of exendin, which can then be visualized by PET.

If successful, this trial will definitively determine if verapamil increases beta-cell mass. Together with the clinical trials completed to date, verapamil appears to show great potential for treating T1D.

### 3.2. Gamma Aminobutyric Acid (GABA)

An orally administrated therapeutic named GABA has gained attention in promoting beta-cell survival and mass [28]. GABA is a major endogenous neurotransmitter of the central nervous system (CNS) that is synthesized from glutamate by glutamic acid decarboxylase (GAD). In an adult brain, GABA is generally considered an inhibitory neurotransmitter, particularly when acting through the GABAA receptor (GABA_A_ R) [29]. Membrane hyperpolarization occurs upon activation of the GABA receptor due to chloride influx through actions on ligand-gated chloride ion channels. Whereas in a developing brain, GABA causes trophic effects through depolarization that normalize neuronal cell proliferation and maturation [30].

**Figure 2:**
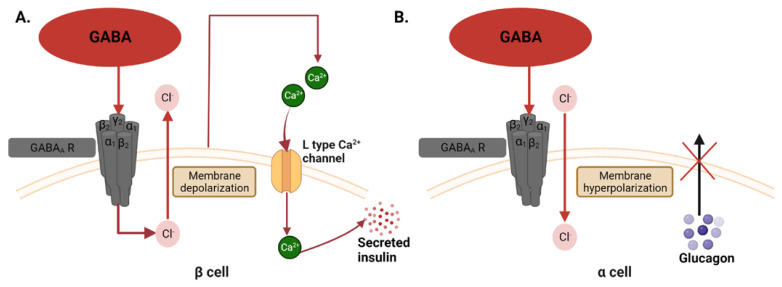
Possible mechanisms of GABA in the beta and alpha cells. (**A**) In the beta cells, GABA binds the GABA receptor and causes an efflux of chloride ions. This leads to depolarization of the membrane, which opens L-type calcium ion channels and allows calcium ions to enter the cell, facilitating insulin secretion. (**B**) In the alpha cell, there is an influx of chloride ions after GABA binds the GABA receptor, and this causes membrane hyperpolarization, preventing glucagon from being secreted out of the cell.

GABA also acts outside of the central nervous system. GABA receptors are found in a variety of immune cells and play a role in immunosuppression, which could impact the development of T1D. As shown in Figure 2A, GABA is also produced by beta cells, where it mediates membrane depolarization and increases insulin secretion [31]. In the alpha cells of islets, GABA is involved in membrane hyperpolarization and results in suppressed glucagon secretion [32], as shown in Figure 2B. Being a mediator of beta-cell survival, GABA is thought to enhance beta-cell proliferation and minimize beta-cell death to ultimately reduce the risk of T1D. A study found that in diabetic mice, GABA also exerts anti-inflammatory effects on the beta cells by providing protection from T cells and macrophages, which contributes to the reduction of insulitis and the recovery of beta-cell mass [29].

Following a phase 1 trial to study the pharmacokinetics of GABA (NCT01917760), a clinical trial (NCT02002130) based on an in vivo study using GABA and GAD-alum was designed to show that GABA alone or in combination with other drugs can be helpful in maintaining beta-cell mass, stimulating insulin, reducing glucagon secretion, and decreasing islet inflammation. This clinical trial also involved testing the insulin dosage among the enrolled participants [33]. In recently published results of this trial, GABA/GAD were administered orally, twice daily, to participants with recent-onset T1D reduced serum glucagon in fasting and meal-stimulated conditions, but did not meet the primary endpoint of maintained C-peptide in the participants [34]. Another clinical trial (NCT03635437), a phase 1, randomized, open-labeled, single-center study, was conducted to investigate the effects and efficacy of the newly developed compound Remygen (GABA compound) therapy, along with insulin intake, for beta-cell regeneration. Among the five participants with no detectable C-peptide levels in the blood, ablated counter-regulatory responses to hypoglycemia were restored by administering subjects 600 mg of GABA for 11 days, which augmented glucagon, epinephrine, and growth hormone in response to hypoglycemia [35]. Future trials will study whether Remygen is potent enough in the regeneration of beta cells and also in controlling hypoglycemic events to reverse T1D [35].

There are several other clinical trials involving GABA in various stages. GABA is also thought to decrease anti-GAD antibodies and cause beta-cell regeneration and is already being tested in a clinical trial along with insulin intake (NCT04375020), but the results are not currently available. Additionally, a phase 2 clinical trial (NCT00529399) has been conducted in which GABA along with GAD-alum were tested. This trial was conducted to investigate endogenous insulin production in new-onset T1D patients. Results showed that GAD-alum, when used in antigen-based immunotherapy, was not effective in overcoming the loss of insulin secretion during T1D. However, insulin usage did not differ among the study subjects [36]. In another phase 2 clinical trial (NCT01561508), GABA, along with insulin, was being tested for the new-onset T1D in children, aiming to help in the insulin production and decrease glucagon release and inflammation, but the clinical trial was withdrawn and has no results. Another randomized, placebo-controlled clinical trial (NCT01781884) was designed to examine C-peptide level differences among those who received GABA treatment and those who received a placebo. This clinical trial also reported insulin intake among the enrolled participants. Another randomized clinical trial (NCT03721991) was designed to test GABA with food supplementation, that aims to improve insulin capacity by turning the alpha cells into beta cells in T1D patients. GABA was tested as an adjunctive therapy with insulin intake. The status of these two trials is unknown. Collectively, these early trials have shown mixed results for GABA-based therapies in the treatment of T1D.

### 3.3. Tauroursodeoxycholic Acid (TUDCA)

TUDCA is a secondary bile acid that belongs to the taurine-conjugated form of ursodeoxycholic acid (UDCA) [37]. TUDCA has proved various unexpected uses in the treatment of liver diseases, neurodegenerative diseases, gastrointestinal disorders, and cardiovascular diseases [37]. Additionally, TUDCA has been shown to be helpful in obesity and diabetes by working on G protein-coupled receptors to help promote the phosphorylation of the downstream target of insulin receptor substrate 1 (IRS1), thereby improving insulin sensitivity [38]. Furthermore, it has been shown to help reduce endoplasmic reticulum stress by improving protein folding and chaperone activity, both of which play significant roles in insulin release from beta cells [39]. TUDCA has been shown to prevent cell death in rat INS-1 cells [40] and to increase insulin secretion from mouse-derived [41] and pig-derived islets [42]. TUDCA has also been shown to reduce the ER stress caused by thapsigargin (an inhibitor of calcium uptake into the ER) and restore the glucose stimulation index of islets [42]. Early TUDCA treatment also showed effective results in restoring visual and retinal function in an STZ mouse model with T1D [43].

**Figure 3:**
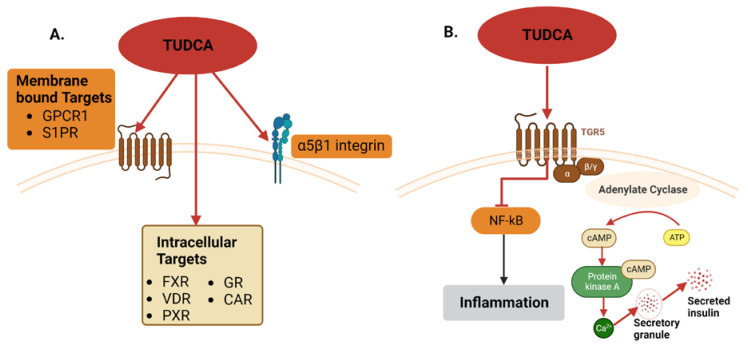
Possible mechanisms of action for TUDCA in the beta cells. (**A**) TUDCA binds to many receptors, including transmembrane receptors GPCR1, S1PR, and a5b1, and intracellular receptors FXR, VDR, PXR, GR, and CAR. (**B**) TUDCA can modulate insulin secretion through the TGR5 receptor. Activating the TGR5 pathway inhibits the pro-inflammatory factor NF-kB and activates adenylate cyclase, which converts ATP to cAMP, leading to insulin secretion.

The exact mechanism of action for TUDCA is still unknown because many receptors and signaling pathways are activated by TUDCA. As shown in Figure 3A, such kinds of bile acids act on both membrane and intracellular receptors. Some of the receptors for TUDCA are sphingosine-1-phosphate receptor 2 (S1PR), α5β1 integrin, and G protein-coupled receptor 1 (GCPR1) or TAKEDA G protein-coupled receptor 5 (TGR5) [39]. TUDCA and other bile acids can also act via intracellular receptors, such as Farnesoid X receptor (FXR), vitamin D receptor (VDR), Pregnane X receptor (PXR), glucocorticoid receptor (GR), and constitutive androstane receptors (CAR) [44].

Out of these receptors, TGR5 seems to be the main receptor for TUDCA in protecting beta cells. As shown in Figure 3B, when this receptor is activated by TUDCA, it generates cAMP, which triggers the signaling pathways. TGR5 has an important role in insulin secretion by pancreatic beta cells when it is activated by ligands such as TUDCA. Along with the cAMP pathway, the PKA pathway is also involved. TUDCA promotes the glucose-stimulated insulin secretion (GSIS) pathway, and in one study it was revealed that its activity was disrupted when both cAMP and a PKA inhibitor were used on mouse islets [41]. It has also been studied that PKA inhibition also disrupts TGR5 activity. Moreover, TUDCA has an important role in the anti-inflammation that occurs via the activation of TGR5 receptors, which in turn activates both the cAMP and PKA pathways, which are involved in GSIS and also in beta-cell survival [39].

There is evidence that TUDCA may have therapeutic benefits in T1D as well. In a longitudinal study of NOD mice, ER stress was shown to precede the onset of T1D [45], suggesting that relieving ER stress could blunt progression early in the disease process. A study the following year showed that indeed, TUDCA administered at the prediabetic stage could reduce diabetes rates in two different T1D mouse models. The beneficial effects of TUDCA appeared to restore ATF6 and sXBP1 signaling, which was aberrant in diabetic mice as well as in pancreatic samples taken from patients with type 1 diabetes [46]. Another preclinical study showed similar beneficial effects of TUDCA on C57BL/6 mice that received intraperitoneal streptozotocin (STZ) for 5 days. In addition, TUDCA was also found effective in regulating the trace elements of the body, such as selenium, in which selenoproteins are composed of and involved in antioxidation, anti-inflammation, and in thyroid hormone metabolism [47]. The relation of selenium and diabetes is controversial, but a study showed that selenium helps in reducing diabetes because it exhibits antioxidant and anti-inflammatory properties [48,49]. Besides reducing the oxidative stress, TUDCA showed a promising result in the selenium distribution in the liver, kidney, heart, and muscle of type 1 diabetic mice, and was also found to be effective in blood glucose levels of mice [50]. These studies concluded that TUDCA is a potential therapeutic agent for the protection of beta cells as well as in insulin stimulation [51].

TUDCA has been clinically approved for liver diseases and gallstones in Europe, and as per the studies, it is also effective in reducing ER stress [38]. A small clinical trial of participants with obesity (NCT00771901) showed that TUDCA can improve insulin sensitivity in liver but not adipose tissue. This clinical trial involved testing TUDCA along with exogenous insulin [38]. This is currently the only published study of TUDCA with respect to diabetes. There is, however, a pilot study to examine the effects of TUDCA administered at a dose of 1750 mg/day for 12 months versus a placebo treatment in participants with early-stage T1D. The status of this phase 2, randomized, triple-masked clinical trial (NCT02218619) is currently unknown. Moreover, there is not enough information on whether this trial involved testing insulin intake and dosage measurements with TUDCA treatment.

### 3.4. Volagidemab

**Figure 4:**
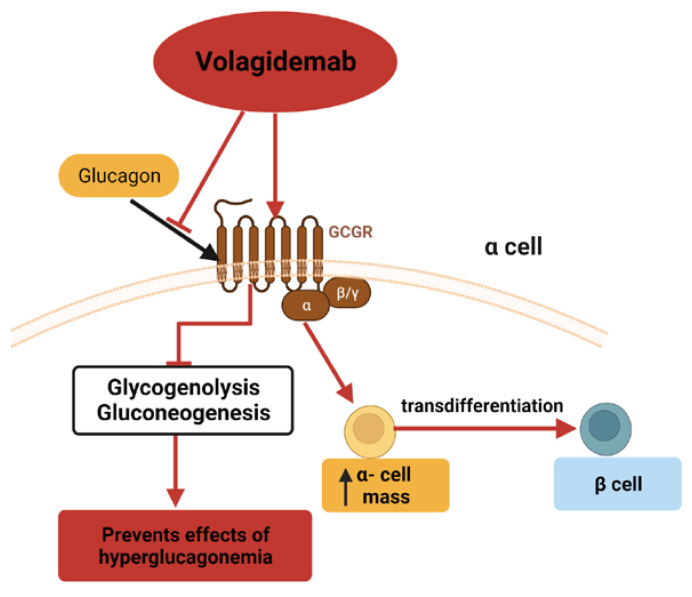
Volagidemab binds the glucagon receptor to prevent glucagon binding, which inhibits glycogenolysis and gluconeogenesis. It also increases alpha-cell mass and allows excess alpha cells to be transdifferentiated to beta cells.

Hyperglucagonemia is present in diabetes, and lowering glucagon levels improves glycemia [52]. When insulin secretion is lost in type 1 diabetes, paracrine inhibition on the α-cells is disrupted, too much glucagon is released, and glucose levels are worsened due to glycogenolysis and gluconeogenesis [52]. As shown in Figure 4, volagidemab (REMD-477) is a humanized glucagon receptor monoclonal antibody (GCGR mAb) that is being investigated for its therapeutic potential in diabetes because it antagonizes the glucagon receptor and may be able to prevent these negative effects of hyperglucagonemia [53].

Volagidemab has been used in diabetic mouse models to prevent elevated blood glucose due to high glucagon action [54]. REMD 2.59 is another human GCGR mAb that differs from volagidemab by one amino acid and has shown reduced gluconeogenesis and blood glucose, improved insulin action and glucose tolerance, enhanced lipid oxidation, protection against diabetic cardiomyopathy, and increased GLP-1 production and L-cell number in mouse models of type 2 diabetes [55,56,57]. REMD 2.59 has also been reported to decrease blood glucose, increase plasma glucagon, somatostatin, and insulin, increase α-cell mass and δ-cell number, which could potentially transdifferentiate into β-cells, and induce β-cell regeneration in normoglycemic and type 1 and 2 diabetic mouse models [58,59,60,61,62,63]. Additionally, this GCGR mAb has been tested for benefits in mouse models of myocardial infarction [64,65]. A different GCGR mAb, REGN1193, has also demonstrated anti-diabetic effects in diabetic mice and monkeys without increasing hypoglycemia in normoglycemic monkeys [66,67].

In addition to these results in preclinical models, volagidemab has been tested in clinical trials. The first study (NCT02715193) was a randomized, double-blind, placebo-controlled, phase 1 trial of a single 70 mg dose of volagidemab that resulted in lowered insulin use one day after injection. The treatment also involved testing insulin intake prior to and after the treatment [68]. Treatment also reduced average daily glucose, increased the percent of time in range, and decreased hyperglycemia without increasing hypoglycemia during days 6–12 after treatment [68]. Another 12-week, randomized, double-blind, placebo-controlled, phase 2 study (NCT03117998) assessed insulin use at week 12 as its primary outcome. The results showed that volagidemab, along with exogenous insulin, reduced insulin needs and improved HbA1c [69]. Additionally, a phase 1 and 2 trial (NCT03919617) in subjects with new-onset type 1 diabetes, administered volagidemab once per week, was completed in May 2022, but the results have not yet been published. Finally, a 12-week, randomized, double-blind, placebo-controlled, phase 2 trial (NCT04779645) is currently recruiting to examine the effects of volagidemab on insulin sensitivity, cardiovascular risk, and ketogenesis, and is expected to be completed in March 2025. The participants remained on insulin therapy throughout the clinical trial. Results of these clinical trials to date suggest that volagidemab has numerous beneficial effects that could potentially treat both type 1 and type 2 diabetes, but critical phase 3 trials have yet to be conducted.

## 4. Conclusions

Among over 3000 clinical trials associated with therapeutic interventions for T1D, the 4 approaches presented here are unique. Rather than developing new forms of insulin or insulin delivery, targeting immune modulation, or repurposing existing T2D drugs, these four potential therapeutics cannot be easily categorized, and each would represent a first-in-class treatment for T1D. The beneficial effects of each drug appear to derive from direct or indirect effects on beta-cell mass, function, and/or survival. If successful, testing of these drugs could be extended to participants at high risk for developing T1D, which could prove to be an even more successful intervention than those reported in this review. While none have been FDA-approved for treatment of T1D to date, the hope is that further success with these drugs in clinical trials will bring more diverse therapeutic offerings in the future.

## Figures and Tables

**Table 1:** Investigational new drugs with potential effects on beta cells.

Drug Name	Target	Key Effects	NCT Number (s)
Verapamil	Voltage-gated calcium channels	Reduced thioredoxin-interacting protein (TXNIP) Reduced calcium influx	NCT02372253 NCT04545151 NCT04615910 NCT04233034
Gamma aminobutyric acid (GABA)	GABA receptor	Membrane depolarization, enhanced insulin secretion, suppressed glucagon secretion. Beta-cell proliferation and protection from cell death	NCT01917760 NCT02002130 NCT03635437 NCT04375020 NCT00529399 NCT01561508 NCT01781884 NCT03721991
Tauroursodeoxycholic acid (TUDCA)	Many receptors, TGR5possibly the main one	Phosphorylation of insulin receptor substrate (IRS) Enhanced GSIS, decreased ER stress	NCT00771901 (type 2 diabetes primarily) NCT02218619
Volagidemab (REMD-477)	Antagonistic mono- clonal glucagon receptor antibody	Inhibition of glucagon action β-cell regeneration (?)	NCT02715193 NCT03117998 NCT03919617 NCT04779645

## Data Availability

Not applicable.
